# Atlas of Venereal and Skin Diseases

**Published:** 1888-08

**Authors:** 


**Atlas of Venereal and Skin Diseases.** Comprising original illustrations and selections from the plates of Prof. M. Kaposi, of Vienna; Dr. J. Hutchinson, of London, and Prof. J. Newman, of Vienna, and others. With original text by PRINCE A. MORROW, A. M., M. D., Clinical Professor of Venereal Diseases, formerly Clinical Lecturer on Dermatology in the University of the City of New York; Surgeon to Charity Hospital, Fasculus IV., V., VI., VIII. New York: William Wood & Co. 1888.

In previous numbers of the JOURNAL favorable notice has been given to this important work on "Dermatology," by Messrs. Wood & Co. The high praise given by the reviewer for the initial numbers is abundantly justified in the excellence of the parts we have just received. The fourth fasciculus contains the following plates:

Plate XVI.—Large papular syphilide, papulo squamous syphilide.

Plate XVII.—Scaly syphilide of trunk and right arm.

Plate XVIII.—Papular and squamous syphilides of palms and soles.

Plate XIX.—Gyrate syphilide, psoriasis, condylomata, lata and condylomata acuminata of genital region.

Plate XX.—Mucus patches of vulva and anal region.

Fasciculus V. contains :

Plate XXI.—Annular syphilide.

Plate XXII.—Chancre of lip, with generalized pustular syphilide.

Plate XXIII.—Large pustular syphilide.

Plate XXIV.—Syphilis cutanea ulcerosa.

Plate XXV.—Rupial syphilide.

Fasciculus VI. contains :

Plate XXVI.—Tubercular syphilide.

Plate XXVII.—Serpiginous syphilide.

Plate XXVIII.—Ulcerative gumnata.

Plate XXIX.—Serpiginous ulcerous syphilide.

Plate XXX.—Syphilis cutanea ulcerosa et vegetans.

Fasciculus VIII. contains :

Plate XXXVI.—Seborrhœa, comedo, milium, sudamina.

Plate XXXVII.—Typhus fever, typhoid fever.

Plate XXXVIII.—Variola-varicella.

Plate XXXIX.—Rubeola, rubella.

Plate XL.—Scarlatina, erysipelas.

The illustrations given by these excellent plates are all that art can furnish. They are life-like and will prove an invaluable aid in the diagnosis and treatment of skin diseases to the general practitioner, whose opportunities are limited in this specialty.

